# Supplementary material for: Are carbon emissions trading and green financial instruments synergistic? -Comprehensive quantitative research based on content analysis
Source: PLoS One. 2024 Mar 7;19(3):e0298601. doi: 10.1371/journal.pone.0298601 (PMC10919601; doi:10.1371/journal.pone.0298601)
Supplement: S1 Data — (ZIP) [file pone.0298601.s001.zip › Data/地级市Z_F工作报告.zip!/─┌├╔╣┼/║⌠┬╫▒┤╢√╩╨/└┤╫╘╣1⁄2╓┌║┼í╛┬φ┐╦╩2╛▌═°í┐_2010.docx]

各位代表：

现在，我代表呼伦贝尔市人民政府向大会作工作报告，请予审议，并请市政协委员和列席会议的同志们提出意见。

一、2009年工作回顾

2009年是新世纪以来我市面临挑战和压力最大的一年。在自治区党委、政府和市委的正确领导下，在全市各族人民的共同努力下，我们深入贯彻落实科学发展观，以"有退有进，美丽与发展双赢"为统领，以"五区"建设为重点，积极应对金融危机带来的巨大挑战，努力克服项目落地难、大宗工业产品价格低迷、财政政策性减收等诸多困难，成功战胜了大风雪、春旱、低温等自然灾害，全力以赴保增长、保民生、保稳定，实现了经济社会逆势而上、平稳较快发展。

综合经济实力进一步增强。全市地区生产总值完成780亿元，增长17.1%。财政总收入完成112.5亿元，增长12.3%；地方财政总收入完成81.8亿元，增长10.2%。固定资产投资完成507.4亿元，增长39.8%。城镇居民人均可支配收入和农牧民人均纯收入分别完成13298元和5606元，增长9.9%和10.8%。工业实施亿元以上项目52个，超10亿元项目20个，实现规模以上工业增加值223亿元，增长28.1%。下大力气推动了各级各类工业园区和产业基地建设，市政府投入3亿元引导资金，实施了园区道路、管网、供电、供水等108项基础设施项目，完成投资26.3亿元，新入园企业达到105家。制定实施了《关于进一步促进中小企业发展的实施意见》，非公有制经济占GDP比重达到45.5％。全面完成了自治区下达的节能减排目标任务。农牧业实现了大丰收，粮食产量达到90.1亿斤，再创新高；牧业年度牲畜头数1552万头只，增长7.2%。在海大两大农垦集团的带动下，全市耕播收综合能力位居全区第一。林区经济实力显著提升，林业总产值增长19.6%，新林区建设日新月异。第三产业快速发展，全社会消费品零售总额完成247亿元，增长19.9％。尤其是旅游业发展迅猛，全年共接待游客800万人次，增长30.9%；旅游总收入完成118亿元，增长26%。全力推进了《天骄•成吉思汗》实景演出、呼伦贝尔民族文化园、牙克石凤冠滑雪场等重点旅游项目和景区建设，海拉尔机场开通航线27条，年进出港旅客55万人次，增幅居全国支线机场首位。金融会展、交通运输、商贸流通、邮电通讯等现代服务业都有较大发展。

发展条件得到明显改善。全市基础设施

建设投资111.6亿元，争取到国家扩大内需资金14.2亿元，一大批公路、铁路、航空、水利、城建和生态建设项目顺利实施。公路建设完成投资56亿元，增长98.6%，占全区公路投资总额的20%。全市首条高速公路绥满国道主干线阿荣旗-博克图-牙克石段顺利推进，海拉尔-阿木古郎、阿木古郎-杜拉尔桥一级公路建设超额完成年度任务，拉布达林-黑山头、根河-满归公路和部分农村公路建成通车。两伊铁路竣工试通车，我市首条地方铁路阿扎铁路开工建设。满洲里机场二期改扩建工程全面完成，成为国际航空口岸，积极推进了5个支线和通勤机场的前期工作。水利建设进程加快，呼伦湖水资源配置及水环境治理工程试运行，红花尔基水库截流蓄水，国际界河护岸和哈布气水电站技术改造等工程进展顺利。实施农村牧区安全饮水工程，解决了15万人饮水安全问题。生态环境持续改善，森林面积和蓄积量持续增长，完成草原围栏建设480万亩、禁牧500万亩、休牧4000万亩、退耕100万亩、黑土区水土流失综合治理5.8万亩。创建国家森林城市工作正式启动，我市被确定为全国11个现代林业示范市之一。全面启动了呼伦贝尔沙区综合治理工程，投入1.57亿元，组织动员10万大军开展生态大会战，治理沙地110万亩，治理区大片流动沙地得到有效控制，投资额度、治理面积和治理效果前所未有。中心城市和小城镇基础设施建设投资30.4亿元，实施市政道路、供水供热、污水垃圾处理等项目229个，城镇功能进一步完善。房屋建设面积687万平方米，完成投资83.3亿元，呼伦贝尔中心城新区建设完成投资11.6亿元，市直行政机关大部分入驻，学校、医院、体育设施即将建成，基础设施基本完善。呼伦贝尔中心城新区市直机关业务用房、哈萨尔斜拉桥、满洲里口岸双子座大厦等一批标志性建筑投入使用，满洲里通湖大道竣工通车。我市荣获"中国人居环境范例奖"。

改革开放迈出新步伐。森工集团企业剥离办社会职能改革首批移交工作全面完成；196家辅业企业组建了新的法人治理结构，划归地方管理。政府机构改革及事业单位岗位设置工作稳步实施。收入分配制度改革逐步完善，公务员津补贴得到规范。医疗、文化体制和集体林权制度改革稳步推进。积极扩大对俄蒙开放，境外投资完成7583万美元，乌兰铅锌矿和诺永达拉果铅锌矿建成投产。呼伦贝尔国际电子商务应用平台正式开通。全年进出口总额完成21亿美元；口岸进出境货运量在金融危机和外贸政策双重不利影响下，仍然实现了正增长，完成2460万吨；实际利用外资8300万美元，增长44.3%。主动走出去招商引资、择商选资，市和各旗市区党政主要领导带队到"长三角"、"珠三角"等发达地区承接产业转移，全年引进国内（市外）资金400亿元，增长18.1%。

各项社会事业全面进步。科技对经济发展的支撑力不断增强，深入实施了科技特派员创业行动，推广普及农牧业实用技术80余项，培训农牧民32.3万人次，建成科技示范园区和特色科技产业化基地12家。各级各类教育全面发展，基本完成了"十一五"教育规划目标。标准化学校建设取得成效。牧区民族教育现代化水平大幅提高。市特殊教育中心竣工，达到全国一流办学水平。高等教育发展迅速，呼伦贝尔学院办学水平进一步提高，内蒙古大学满洲里学院建成招生，职业技术学院建设进展顺利。全面启动了中小学校舍安全工程。大力发展民族文化事业，五彩呼伦贝尔儿童合唱团等"五张文化名片"影响力进一步提升；博物馆事业全面进步，呼伦贝尔民族博物馆正式对外开放，人口较少民族文化遗产保护工程扎实推进。文学、艺术、出版等文化领域成果丰硕。全面加强公共卫生服务体系建设，积极应对并科学防控甲型H1N1流感疫情，实施了97个基层卫生服务体系建设项目，新型农村牧区合作医疗参合率达到95.4%，加大了监管力度，确保了食品药品安全。全市城镇新增就业2.6万人，农牧民转移就业6.2万人，高校毕业生实现就业1.3万人。进一步扩大社会保险覆盖范围，完善社会保障体系，基本养老保险达到40.4万人、失业保险达到36.4万人、医疗保险达到117万人、工伤保险达到19.6万人、生育保险达到17.7万人，全部超额完成了年度目标任务。积极推进扶贫工作，解决了10万贫困人口温饱问题。深入实施人才强市战略，首次出台高层次紧缺人才引进实施办法，引进硕士研究生、副高职称以上高层次人才513人。人口计生工作全面加强，低生育水平持续稳定。体育事业蓬勃发展，48人参加十一届全运会，取得一金一银一铜的优异成绩；成功举办了首届全市老年人运动会。广播电视公共服务体系建设全面加强，广播电视覆盖率分别达到90.8%和92.5%。民族宗教、外事侨务、信息化、国土、工商物价、接待联络、档案史志、红十字、供销、人防、气象、地震、质监、老龄、残联、金融保险等工作都有了新进步。

人民群众得到更多实惠。"六个一"幸福指数工程扎实推进。民生事业投资达57亿元，投资额度和惠及面再创历史新高。市直和各旗市区行政事业单位年人均增加津补贴7428元和6589元，20万城镇低保对象年人均补助提高了396元，9万农村牧区低保对象年人均补助提高了313元，8.8万地方企业离退休人员年人均增加养老金1404元，森工集团在岗职工年人均增加工资1200元。重点加大了对人口较少民族的扶持力度，人口较少民族群众年人均收入提高了1000元以上。敖鲁古雅鄂温克猎民户均住宅面积由50平方米提高到88平方米。鄂伦春猎民生产性收入年人均增加2500元。

我们承担的自治区10件为民办实事项目圆满完成。市政府在市二届三次人代会上向全市各族人民承诺的10件为民办实事项目全部兑现。一是实施了总投资27.5亿元、209.5万平方米的廉租房和棚户区改造等一系列保障性住房建设，惠及12.7万人。二是孤老残幼福利家园主体工程全部竣工。三是呼伦贝尔市人口较少民族传统文化保护工程正式启动，完成了年度任务。四是实现了牧区蒙语广播节目全覆盖，为所有牧民家庭配发了收音机。五是设立了全市道德模范奖励助困基金，出台了《呼伦贝尔市道德模范奖励助困基金实施办法》。六是新增了10条公交线路，新增和改建485个公交设施，有效解决了群众出行难问题。七是改善了80所农村牧区寄宿制学校生活设施，惠及学生1.5万人。八是安排专项补贴，帮助55名贫困家庭患先天性心脏病儿童进行了康复手术。九是为全市独生子女、农牧区双女结扎户女孩和计生干部交纳了人身意外伤害保险，做到了全覆盖。十是农村五保供养标准每人每年提高了200元，惠及5511人。10项公共公益工程稳步实施。沙区综合治理、工业园区基础设施建设、中心城区主街道综合治理、农村牧区文化站和卫生室建设全部完成。旗市区污水和垃圾处理工程责任地区全部开工，建成2座污水厂和4座垃圾无害化处理场。呼伦贝尔中心城新区中小学、呼伦贝尔中心城新区河东基础设施、白音哈达草原景区、民兵预备役基础设施改造、海拉尔北出口景观大道等工程有序推进。跨年度公共公益工程进展顺利。水源地改造主体工程竣工。市职业技术学院一期主体工程竣工。市图书馆、体育场、海拉尔火车站改造、"三少"民族自治旗通乡公路、满洲里新国际货场等跨年度工程进展顺利。

精神文明和民主法制建设得到加强。精神文明建设亮点纷呈，成功召开了全市第六届两个文明建设经验交流会，圆满承办了全区社会治安综合治理工作会议，提炼形成了"博大、至诚、和美、共赢"的呼伦贝尔城市精神。全面加强政府自身建设，深入开展"三抓一建"活动，政府公信力和执行力进一步增强，人民满意度进一步提高。自觉接受人大依法监督和政协民主监督，与各民主党派、工商联和无党派人士联系更加紧密，办理人大代表议案和建议140件，政协委员提案213件。积极支持工青妇等群众团体工作。审计监督成效进一步显现。积极开展优化发展环境年活动，廉政建设和反腐败斗争更加深入。"安全稳定呼伦贝尔"创建活动成效突出，圆满完成了建国60周年安全维稳和庆祝活动。综治工作首次获得全国优秀地市称号。深入开展矛盾纠纷排查化解、党政主要领导大接访、信访积案化解年等活动，信访形势明显改善。安全生产形势继续好转，各项控制指标均达到历史最好水平。边防安宁稳定，双拥工作成效突出，满洲里边防检查站被国务院、中央军委授予"爱民固边模范边防检查站"的荣誉称号，呼伦贝尔市边防委员会被国家边海防委员会、国家人力资源和社会保障部、总参谋部、总政治部联合表彰为全国边海防先进集体。

各位代表，在过去的一年里，我们克服了各种困难，保持了经济社会平稳较快发展的良好势头，尤其是在重视和改善民生上，取得了突出的成效。这是自治区党委、政府和市委正确领导的结果，是市人大、市政协有效监督、鼎力支持的结果，是全市各族干部群众解放思想、奋力拼搏的结果，是社会各界大力帮助、团结协作的结果。在此，我代表呼伦贝尔市人民政府，向全市各族干部群众，向所有关心支持呼伦贝尔发展的同志们、朋友们，表示衷心的感谢和崇高的敬意！　　在看到成绩的同时，我们也要清醒地认识到在发展中还存在很多困难和问题：一是经济总量小，结构不尽合理，新的优势产业还需培育，区域间发展不平衡，中心城市吸引力和辐射力不强。二是经济外向度不高，对发达地区资金、技术、人才、产业的吸引力不强，自身发展的内生力不足，非公经济有待进一步壮大。三是农村牧区基础薄弱，农牧业产业化水平不高，分散的农牧业生产方式不适应现代农牧业发展的需要。四是"原字号"工业产品比重大，产业链短，产业结构优化升级有待加强。五是生态建设与保护任务仍然繁重。六是居民收入增长与经济增长不协调，林区、人口较少民族群众生活还比较困难，就业和再就业压力仍然很大，一些因素仍然影响社会稳定。七是政府自身建设需进一步加强，还不同程度地存在着官僚主义、形式主义、本位主义、消极腐败等现象。上述问题，必须引起高度重视，认真加以解决。

二、2010年主要任务

2010年，是"十一五"规划的最后一年，也是为"十二五"规划启动实施奠定坚实基础的关键一年。我们将迎来世界经济企稳回升的良好环境，迎来国家深入实施东北振兴、资源型城市转型、大小兴安岭林区生态保护与经济转型以及中俄蒙合作战略升级等一系列难得的机遇，迎来重大项目投产达效的快速发展期。同时，我们也面临着金融危机余波尚在、项目审批难度增大、结构性矛盾依然突出等诸多不利影响。当前，呼伦贝尔已经全面进入了富民强市、科学发展的关键时期，我们肩上的担子更重、责任更大。我们一定要始终坚持发展是第一要务的思想不动摇，继续保持经济发展的良好势头，在转机中抢抓先机，在发展中再造优势，团结一心，勇克时艰，努力开创经济社会发展的崭新局面。

今年政府工作的总体要求是：全面贯彻党的十七届三中、四中全会、全区经济工作会议和市委二届十五次全委会议精神，以科学发展观为统领，以富民强市为目标，以做大经济总量、调整经济结构、转变发展方式为主要任务，以"五区建设"为重点，深入实施"有退有进，美丽与发展双赢"战略，大力推进新型工业化、农牧业产业化、城镇化和新型边境区域合作进程，更加注重民生和社会事业，更加注重生态和城乡基础设施建设，更加注重改革开放和优化发展环境，更加注重精神文明、民主法制和政府自身建设，努力实现经济社会又好又快发展。

今年全市经济社会发展的预期目标是：地区生产总值增长17%，达到912亿元；固定资产投资增长25%，达到634亿元；财政总收入增长20%，达到135亿元，地方财政总收入增长20%，达到98亿元；城镇居民人均可支配收入增长17%，达到15560元；农牧民人均纯收入增长15%，达到6450元。城镇登记失业率控制在4.2%以内。人口自然增长率控制在5.13‰。全面完成自治区下达的节能减排任务。

实现上述目标，今年重点抓好七项工作：

（一）继续做大总量优化结构，提高工业经济的质量和效益

巩固提升优势特色产业，培育发展新兴产业，以园区和项目建设为重点，全力扩大工业经济总量、优化工业经济结构。

做大总量优化结构。工业固定资产投资达到400亿元，增长37 %；规模以上工业增加值达到267亿元，增长20%。在优势特色产业发展方面。要以煤、电、油、化、有色金属为重点，大力提高产品延伸和就地加工转化水平。煤炭就地转化率要达到31%。全力推进与东北电网的联网送出工程和呼伦贝尔电网增强性改造工程，扩大外送及市域内的供电能力。切实提高有色金属产业的延伸水平，形成分散采矿、定点选矿、集中冶炼的组合模式，就地转化率达到100%。扩大进出口产品加工规模，做大满洲里进出口产品加工基地。提高农畜产品加工深度，农畜产品加工转化率达到60%。在新兴产业培育方面。要大力发展低碳经济，积极规划建设风能、太阳能发电项目，在建风电装机要达到50万千瓦，力争启动2×1万千瓦太阳能电站项目。大力发展装备制造业，争取年内投产锦化机装备制造业项目，力争开工年产3000辆压缩空气动力旅游区专用车项目。积极支持同联呼伦贝尔项目建设。大力推进工业化与信息化融合，提高企业研发、生产、管理和销售的信息化水平，加大信息产业项目的招商引资力度，推动电子信息产品制造业发展。

强力推进工业重点项目。项目建设是工业经济发展的核心。既要重视扩能升级项目，也要重视引进新的项目；既要重视大项目，也要重视中小项目；既要重视项目的建设，也要重视项目的储备。今年，市本级实施重点工业项目82个。煤炭产量由5300万吨增加到6000万吨，开工建设谢尔塔拉露天矿、扎尼河露天矿、宝日希勒2号露天矿等项目。电力装机由280万千瓦增加到800万千瓦，实现360万千瓦外送电源、热电联产和风电项目并网发电，新开工汇流河2×20万千瓦热电。积极推进京能源2×30万千瓦热电项目前期工作。化工要确保金新80万吨尿素、大唐50万吨尿素项目试车生产，力争开工国电新左旗50万吨尿素、华电新左旗40万吨乙二醇和东能陈旗50万吨乙二醇等项目，加快推进扎赉诺尔60万吨甲醇项目建设。确保驰宏20万吨铅锌冶炼项目试运行。继续推动大庆石油接续性开发，境内外石油产量力争达到100万吨。

继续加大对工业园区的支持力度。要继续强抓工业园区建设不动摇，做到投入力度不减、工作氛围不降，把工业园区打造成既是产业集聚区也是产业延伸区，既是工业区也是新城区，既是财政贡献区也是增加就业区。进一步完善园区总体规划，加大对市和各旗市区重点园区的支持力度，全面达到"四通一平"标准。每个工业园区至少要引进2-4个投资亿元以上的工业项目，园区工业增加值要超过170亿元，占到全市工业增加值的60%以上。加快推进呼伦贝尔经济开发区两村移民工作。

大力发展非公有制经济。要充分认识非公有制经济在我市不可替代的作用，着力解决非公企业在发展中存在的低小散的问题，用足用活上级政策，制定出台我市扶持非公企业发展的政策措施，促进非公企业快速发展，让非公经济唱主角、挑大梁、坐正席。加大金融扶持力度，引进股份制银行，完善担保体系，切实帮助非公企业解决融资难的问题。各级各部门都要为非公企业发展创造良好环境，营造全社会共同支持非公企业发展的良好氛围。积极鼓励和引导非公企业围绕优势特色产业搞延伸，围绕重点项目搞协作，围绕大型基地搞配套，促进非公企业进入园区集群化发展。全力打造本土企业家队伍，促进非公企业健康快速发展。今年全市非公有制经济占GDP比重提高4.5个百分点，达到50％。

全面抓好节能减排。大力发展循环经济，延伸产业链条，构建节能型工业体系。加强行业节能，落实好年耗能5000吨标准煤以上各级重点能耗企业的节能责任目标。加大减排工作力度，全面加强结构减排、工程减排和管理减排，开展重点污染源治理，积极淘汰落后产能。二氧化硫排放总量控制在10万吨以内，化学需氧量排放总量控制在1.8万吨以内，全面完成"十一五"节能减排任务。

（二）夯实农牧业基础地位，促进农牧民持续增收

不断提高农牧业综合生产能力。第一产业增加值达到167亿元，增长8%，粮食总产量达到100亿斤以上，牧业年度牲畜头数稳定在1500万头只。要围绕粮食增产工程，加强农田水利基本建设，大力发展节水灌溉，继续实施中低产田改造和黑土区综合治理工程，加强节水灌溉人工草牧场和饲草料基地建设。全年新增节水灌溉面积25万亩，达到291万亩，新增灌溉饲草料地面积3.2万亩，达到25.3万亩。农业要以市场为导向，增加玉米、水稻等高产作物种植面积，稳定大豆、小麦等政策扶持作物种植面积，减少低质、低产作物种植面积；畜牧业要增大减小、少养精养、规模化养殖。大力开展动植物新品种、农牧业实用新技术和农牧业科技新产品研究创新，积极推广免耕播种、配方施肥等实用技术，全面提高机械化作业和粮食单产水平。着力加强动植物防检疫体系建设，确保无大疫。

深化农村牧区经营体制改革。按照市委一号文件要求，以土地草场流转为突破口，以新型经济合作组织为载体，以发展现代农牧业为方向，以"三改一建设"为主要内容，推动土地规模经营，农区年内流转土地270万亩，全市土地规模经营要达到1000万亩。在牧区选取4-8个草牧场流转试点，典型示范，在2-3年内推开。提高农牧民市场经济意识，规范扶持种植、养殖、加工和购销类经济合作组织发展，经合组织助农增收率要达到20%以上。

下大力气抓好农牧业产业化。坚持引进与培育相结合，做大做强龙头企业。在抓好现有乳、肉、豆、薯等龙头企业发展的同时，重点扶持具有区域带动作用的生猪、大鹅、玉米加工等大型龙头企业，瞄准国内知名企业，加大招商引资力度，千方百计扶持、壮大农牧业产业化龙头企业的规模和实力。全市销售收入百万元以上加工企业增加值要达到55亿元，销售收入168亿元，均增长40%以上。狠抓主导产业和特色产业的发展，优化农牧业区域布局，建设大型的农牧业科技园区、绿色生态园区，打造现代农牧业示范带，大力推进奶牛、肉牛、生猪、家禽规模化、科学化养殖小区建设，建立稳定的农畜产品生产基地。进一步探索和完善利益联结机制，促进农牧业产业化健康发展。

扎实推进新农牧林区建设。继续提升新农牧林区建设试点村（嘎查）和样板村建设水平，扩展新农牧林区建设重点推进村，形成联片创建新格局。引导扶持村（嘎查）培育1-2个支柱产业，试点村、样板村和重点推进村农牧民人均纯收入要高于全市农牧民人均纯收入500元以上。通过村级组织"一事一议"财政奖补政策，加大农村牧区基础设施和公共公益设施建设力度，启动农村牧区危房改造工程。深入实施"百村美化"工程，大力整治和改善农村牧区环境状况。重视牧区工作，实施游牧民定居工程，提高嘎查（村）干部的报酬待遇，在加快牧区基础设施建设上给予倾斜，落实好划区轮牧补贴试点，加大对牧区购置良种牲畜、牧业机械的补贴力度。全力承办好全区第三届新农村新牧区精神文明建设现场经验交流会。

多措并举提高农牧民收入。全面落实国家各类政策性补贴，确保全部到位；进一步优化种养业结构，以调整促进增收；全面提高农牧业科技含量，以科技带动增收；大力发展劳务经济，以劳务输出拉动增收；全面抓好农业保险，实现保障增收；引导农牧民多渠道投资，增加财产性收入。

（三）突出旅游业带动作用，全面提高服务业发展水平

旅游业。要开展好"旅游景区建设年"活动，继续抓好呼伦贝尔民族文化园、白音哈达草原景区、哈克遗址博物馆、满洲里新国门、敖鲁古雅、甘珠尔景区、宝东山祭祀、达斡尔民族园、东北抗联英雄纪念园等重点景区建设，启动蒙古之源•蒙兀室韦和拓跋鲜卑两个大型民族文化园、南木鄂伦春猎民部落、喇嘛山景区、鄂温克百公里景观带等景区建设，抓好红花尔基森林旅游景区的提升改造。推动具有区域特色的家庭旅游业发展。要切实提高接待能力，积极引进国内外品牌连锁酒店，引导社会投资建设经济适用型宾馆、社会旅馆和家庭公寓，鼓励对闲置办公楼房进行宾馆式改造，确保中心城区床位达到2万张。加大旅游大交通建设力度，国内外航线力争达到30条。利用中央电视台广告和我市特色节庆活动，重点针对北京、东三省、"长三角"和"珠三角"等客源市场，开展旅游宣传促销。开发旅游产品，强化旅游行业管理，规范旅游市场秩序。全年旅游人数突破1000万人次，增长25%；旅游业总收入完成142亿元，增长20%。

物流业。重点支持海、满、牙、扎等地区打造区域性物流中心，促进跨区物流发展。加快推进新型农牧林区物流网络建设，鼓励具备条件的乡镇发展面向生产和生活的快捷便利物流业, 充分利用"双百市场工程"和"万村千乡市场建设工程"，做大做强各类专业批发市场，重点抓好粮食、牲畜和特色农畜产品市场建设，改善农牧林区消费环境，促进城乡统筹发展。继续加大"家电下乡"工作力度，积极推进汽车以旧换新和汽车、摩托车下乡工作，让更多农牧民受益。

金融保险业。积极发挥金融对地方经济发展的支持作用，年内引进内蒙古银行、浦发银行、盛京银行、招商银行入驻我市。要扩大市本级担保公司规模，积极推动各旗市区小额贷款公司建设。加快村镇银行建设步伐。加强社会征信体系建设，改善金融生态环境，为金融业发展创造良好条件。要加强政策引导，发挥保险保障和融资功能，切实提高保险服务水平和防范风险能力。

房地产业。要运用多种手段加强市场调控，培育完善房地产交易市场，精心举办房交会，促进房地产市场健康发展。年内完成投资90亿元，房屋建设面积超过700万平方米。

全面推动商务会展、交通运输、邮电通讯、研发咨询、供销仓储等现代服务业加快发展。

（四）继续加大生态保护与基础设施建设力度，不断改善城乡发展条件

全面加强生态市建设。生态环境是我市经济社会发展的前提和基础。今年要继续以沙区综合治理为重点，统筹抓好农区、牧区、林区、垦区、矿区生态建设，继续完成退耕100万亩、草原建设500万亩、休牧5000万亩的生态建设任务，牧区牲畜头数稳定在400万头只，森林采伐量控制在200万立方米左右。充分发挥我市得天独厚的碳汇资源优势，着力把呼伦贝尔打造成为祖国北疆重要的森林草原碳汇基地。沙区综合治理要做到投入、任务、氛围"三不减"，组织动员全社会力量，开展大规模沙区综合治理大会战，确保再完成治理沙地100万亩，坚决遏制沙化势头。要加大生态移民力度，积极探索移民后产业发展新途径，完善生产生活条件，拓宽增收渠道，做到搬得出、稳得住、能致富，年内完成生态移民650户。要加大沙区治理后期管护力度，抓紧出台实施沙区治理后期管护办法，确保治沙效果得以巩固。

加快城镇化进程。按照"做大中心城市、构建都市圈，打造小城镇，提高城镇化水平"的总体要求，进一步加快城镇化进程，全市城镇化率提高到67.5%。要切实提高城市规划水平，把城市规划作为城市发展的中心环节，用世界的眼光、用前瞻的目标，抓好城市总体规划和区域规划。要坚持市区共建、市区一体，突出抓好以海拉尔为中心辐射周边的呼伦贝尔中心城发展。今年要全面启动呼伦贝尔中心城新区河东部分建设，精心打造以"一湖三馆一院"为核心的呼伦贝尔文化产业园区，加快推进海拉尔火车站站房和站前环境整治工程建设。加快满洲里新区建设。统筹抓好各旗市区所在地和重点城镇建设，着力打造一批有民族特色，有产业支撑的城关镇、口岸城镇和旅游城镇。要不断完善城镇功能，抓好投资35亿元的232个城镇基础设施项目建设，尚未建成污水处理厂的6个旗市年内全部建成，尚未建成垃圾无害化处理场的7个旗市要全部开工建设。要加强城市治安、市容卫生、交通秩序综合整治和水电气热配套服务，提高城市管理水平。中心城区给水改扩建工程要确保6月底投入使用。继续实施好棚户区改造、廉租房建设和游牧民定居等工程，计划投资33.1亿元，建设面积达到245.5万平方米。

加快基础设施建设。公路要抓好阿荣旗-博克图-牙克石高速公路、海拉尔北出口景观大道等续建项目，全力争取牙克石-海拉尔高速公路、满洲里-阿拉坦额莫勒-阿木古郎一级公路早日开工建设。积极推进海拉尔-拉布达林、拉布达林-根河、碾子山-博克图一级公路项目的前期工作。继续抓好通乡通村、林区和旅游景区公路建设。全年确保完成公路建设投资57亿元，力争完成67亿元，增长20%。铁路要加快推进阿扎铁路建设，争取开工满伊铁路、海拉尔-拉布达林-黑山头等新的铁路项目，积极推进阿荣旗-莫旗-讷河、扎兰屯-柴河-阿尔山、室韦-莫尔道嘎铁路前期工作，争取哈齐客运专线延伸至呼伦贝尔。民航要抓好海拉尔东山机场停机坪扩建，全力推进扎兰屯、阿里河、根河、莫尔道嘎、尼尔基支线和通勤机场项目前期工作，争取早日开工建设。水利要全面加快水利枢纽工程建设，确保红花尔基水利枢纽工程完工，扬旗山水利枢纽复工建设；做好尼尔基灌区项目审查报批和扎敦河、晓奇水库项目前期工作。继续实施农村牧区安全饮水工程，解决9.5万人饮水安全问题。

（五）深化改革开放和区域合作，增强发展的动力与活力

扎实推进改革。政府机构改革要理清职责，整合机构，科学制定《三定》规定，确保按时完成。财政改革要大力推进科学化、精细化管理，继续深化国库收付制度改革、非税收入收缴制度改革、政府采购制度改革，规范国有资产管理，对市本级投资的部分公共公益项目开展代建制试点探索，切实解决工程建设"超规模、超标准、超概算"的问题。文化体制改革要大力推进文化领域资源整合，加快构建有利于科技与文化产业相结合的体制机制。医疗卫生体制改革要全面启动实施促进基本公共卫生服务逐步均等化项目，实施基本药物制度，积极探索城乡基层公立医疗卫生机构运行机制改革。继续支持森工、煤炭、农垦等国有大企业建立现代企业制度。

全面扩大开放。要抓住全球经济形势企稳回升和《中国东北地区同俄罗斯远东及东西伯利亚地区合作规划纲要》全面实施的重大机遇，巩固发展与俄蒙毗邻地区政府间友好关系，促进经贸、旅游、文化和教育等领域的合作，全面提升开放水平。阿日哈沙特口岸国际性常年开放和额布都格口岸正式对外开放。进一步加大境外资源开发力度，积极推进俄罗斯诺永达拉果铅锌矿、别列佐夫铁矿和蒙古国乌兰铅锌矿等境外资源开发项目建设。全力推进本地进出口产品生产、加工和销售基地建设，帮助有条件、有基础的外贸企业做大做强。进一步加大择商选资的力度，主动承接"长三角"、"珠三角"和环渤海经济圈等发达地区的产业转移。全年引进国内（市外）资金达到460亿元，增长15%。

加强区域合作。积极融入东北振兴，全面加强与东三省特别是黑龙江省的合作，建立健全沟通协调机制，在发展规划、基础设施、工业项目、旅游协作、对外开放和统一市场建设等方面加强对接，打造"哈大齐呼"经济协作区，使我市成为东北地区的战略资源接续基地、产业转移延伸基地和国家向北开放前沿阵地。

（六）高度重视和改善民生，大力推进社会事业发展

千方百计扩大就业。建立健全促进就业的长效机制，重点加强对城镇下岗失业人员、高校毕业生和需要转移就业农牧民的扶持，确保城镇新增就业2.5万人，农村牧区劳动力转移就业7万人左右，动态实现每个有就业能力和愿望的"零就业"家庭至少1人就业。大力扶持劳动密集型小企业发展，重点抓好"创业园区"、"创业孵化基地"建设，大力促进创业带动就业。积极开展对"4050"人员、"零就业家庭"、零就业转移家庭"及其他就业困难群众的就业援助，努力实现充分就业，提高收入水平。

切实提高社会保障水平。进一步建立健全覆盖城乡的社会保障体系，提高社会保险待遇水平，突出抓好社会保险政策完善和扩面征缴工作，全面启动"三少"民族自治旗国家级和自治区级新型农村牧区社会养老保险试点工作，做好城镇职工和居民基本医疗保险的提标扩面工作，基本解决所有关闭破产企业退休人员和困难企业职工参加医疗保险问题。完善社会救助体系，稳步提高城乡低保标准和补助水平，抓好贫困家庭大学生救助工作，加快推进孤老残幼福利家园建设。强化劳动监察执法，严格落实最低工资标准，建立企业职工工资正常增长机制，维护劳动者与企业合法权益。市直行政机关和事业单位人均月增加津补贴500元，各旗市区根据财政支付能力也要有不同程度的增长。精心组织实施整村推进、产业化扶贫工程，积极争取自治区集中连片开发项目，解决10万贫困人口的温饱问题。重视发展公益慈善事业，全面做好残疾人保障体系、服务体系建设和"全国无障碍城市"验收工作。做好老龄工作。如期完成妇女和儿童发展纲要的各项达标任务。

办好10件实事。今年市政府继续为民办10件实事：一是实施中小学校舍安全工程。改造、加固、新建中小学校舍124.5万平方米，为全市中小学生提供最牢固、最安全、最放心的学习环境。二是为集体企业下岗失业"4050"人员发放社会保险补贴，惠及3.8万人。三是实施农村牧区危房改造工程，改善2300户农牧民居住条件。四是改造农村牧区电网，降低农牧民用电价格，实现广大农牧民期盼多年的同网同价，惠及全市90万农牧民。五是解决大学生就业难问题，多渠道为全市大学生提供1.35万个就业岗位。六是实施以农村牧区为重点的改厕工程。改造新建厕所1.5万个，解决农牧区、城镇和旅游景点厕所标准低、条件差的问题。七是较大幅度提高全市村（嘎查）干部报酬待遇，进一步调动广大村（嘎查）干部的工作积极性。八是扶持鄂伦春、达斡尔、鄂温克"三少"民族发展。重点启动"三少"民族自治旗新型农村牧区社会养老保险试点，支持南木鄂伦春民族乡产业发展，继续加大扶持人口较少民族发展和文化遗产保护专项资金的投入。九是实施市民综合服务体系场馆建设工程。建设功能完备、环境舒适、利民便捷，集人才交流、公共就业服务、社会保障、行政审批等综合服务功能为一体的市民大厦。十是免费为牧区育龄妇女提供健康体检，惠及1.5万人。

实施10项公共公益项目。一是实施农牧业产业化重点工程。二是扩建海拉尔东山机场停机坪。三是建设呼伦贝尔精神卫生中心。四是建设呼伦贝尔孤老残幼福利家园休闲区。五是建设呼伦贝尔新闻中心。六是建设呼伦贝尔艺术馆。七是建设呼伦贝尔科技馆。八是建设呼伦贝尔城市展览馆。九是建设蒙古之源•蒙兀室韦民族文化园。十是建设拓跋鲜卑民族文化园。积极推动科技创新。以建设创新型呼伦贝尔为目标，加快区域科技创新体系建设，规划建设呼伦贝尔市科技创业服务中心。以科技项目为引导，推进科技成果转化。完善以市场化为导向的产学研相结合的科技创新体制，全面实施知识产权、科技名牌战略，鼓励企业研发具有自主知识产权和核心技术的专利产品。

优先发展教育事业。保持教育投入稳定增长，全面实施素质教育，大力推进各级各类教育协调发展和教育公平。重点加强义务教育标准化学校建设。加大高中阶段教育普及力度。加快发展职业教育，加强中等职业教育基础能力建设，全面完成职业技术学院工程建设任务，确保秋季招生。确保呼伦贝尔学院顺利通过教育部本科教学工作合格评估，支持内蒙古大学满洲里学院加快发展。优先发展民族教育，加强"双语"师资队伍建设。鼓励引导民办教育健康发展。重视特殊教育发展。重点抓好校舍安全工程，确保完成年度工作任务。

大力发展卫生事业。加强基层医疗卫生服务体系建设，完善新型农村牧区合作医疗制度，努力促进基本公共卫生服务逐步均等化。继续做好甲型H1N1流感联防联控工作，不断增强控制重大疾病和应对突发公共卫生事件的能力。大力推进蒙中医药事业发展，抓好市蒙医院和精神卫生中心建设。加快发展社区卫生服务，积极推进乡镇和村（嘎查）标准化卫生院室建设。抓好食品和药品安全专项整治，确保群众饮食用药安全。继续做好人口计生工作，深入开展出生人口缺陷干预和生育关怀行动，提高出生人口素质，稳定低生育水平，人口出生率控制在11.6‰以内。

加快发展文化事业。认真贯彻落实国务院《关于进一步繁荣发展少数民族文化事业的意见》，加快民族文化大市建设步伐。充分利用五彩呼伦贝尔儿童合唱团等"五张文化名片"，形成文化品牌效应。积极发展文化产业，重点打造以"一湖三馆一院"为重点的呼伦贝尔中心城文化产业园区。改善公共文化服务基础设施条件，确保呼伦贝尔大剧院投入使用。全面完成乡镇（苏木）综合文化站工程建设。继续推动博物馆市建设，推动博物馆等公益性文化单位免费向社会开放。抓好文化普查和人口较少民族文化遗产保护。认真贯彻落实国务院《全民健身条例》，促进体育事业全面协调发展，积极参加自治区第十二届运动会和第六届少数民族传统体育运动会。全面加强广播电视工作。不断提高广播电视制作播出能力，继续推进"村村通"工程建设，完成全市9200户的直播卫星接收系统和无线覆盖工程建设任务。支持通信业务建设。

全面做好人事人才工作。进一步完善促进大中专毕业生就业的政策体系，落实好"三支一扶"等选拔高校毕业生到基层工作计划。突出做好创新型高层次人才和国外智力引进、企业和农牧林区人才资源开发等工作，强化"421"人才工程建设，深入实施服务新农牧林区建设"1+1"人才工程，进一步落实服务大企业大项目"1+3"工作机制。积极推进人力资源市场建设。

认真做好统计、审计、国土、民族宗教、档案史志、气象、地震、质监和对外联络等工作。

（七）维护社会和谐稳定，加强精神文明、民主法制和政府自身建设

全力维护社会稳定。落实"稳定是第一责任"的总体要求，建立社会稳定风险评估机制。启动"平安呼伦贝尔建设"活动，严格落实维稳工作责任制，全面加强社会治安综合治理，严厉打击违法犯罪活动。高度重视信访工作，认真解决信访突出问题，积极做好群体性事件预防处置。完善应急管理体制机制，提高预防和处置突发公共事件能力。重视和加强国家安全工作，加强边境管控力度，确保边疆安全稳定。抓好国防动员和人防体系建设，深入开展双拥共建和优抚安置工作，巩固军政军民团结大好局面，全面做好迎接全国、全区双拥模范城检查验收和命名表彰工作。

切实加强安全生产。牢固树立安全发展理念，健全各项安全生产制度，认真落实安全生产责任制，切实抓好重点行业、重点地区、重点企业的安全治理，加大对煤矿、危化、交通和人员密集场所的安全监管力度。加强森林草原防火和城市消防工作。抓好救援体系建设，提高施救能力。

加强精神文明建设。大力加强社会主义核心价值体系建设。巩固学习实践科学发展观活动成果，不断增强干部群众科学发展的自觉性和坚定性。继续大力弘扬呼伦贝尔城市精神，不断增强和谐呼伦贝尔建设软实力。组织开展全市第二届道德模范评选活动。额尔古纳市和新左旗要全力承办好全市第七届两个文明建设经验交流会。

加强民主法制建设。认真执行人大及其常委会的各项决议、决定，自觉接受监督。积极支持政协履行政治协商、民主监督、参政议政的职能。认真办理人大代表议案、建议和政协委员提案，主动听取各民主党派、工商联、无党派人士及各人民团体的意见和建议。大力支持工青妇等群团组织开展工作。深入开展普法教育，做好法律援助工作。完善基层民主管理制度，提高村民自治水平。加强社区建设和管理，更好地发挥社区在就业、社保及维护稳定方面的重要作用。

建设人民满意政府。要按照大力提高政府执行力、创新力、公信力和凝聚力，打造阳光政府、建设人民满意政府的要求，严格依法行政，按照法定权限和程序行使权力、履行职责、规范行为。深化行政审批制度改革，推行行政审批权公开透明运行，协调推进以行政审批服务中心为平台的政务服务体系建设。做好政务公开工作，保障群众对政府工作的知情权、参与权和监督权。要加强作风建设，大兴调查研究、真抓实干之风，切实解决好关系群众切身利益的突出问题。要狠抓落实，确保政令畅通。加强廉政建设，加大对重点资金、重点项目的审计监督和行政监察，要继续压缩政府部门经常性项目支出，严格控制会议、差旅、出国、接待等费用。各级干部特别是领导干部要模范遵守廉洁从政的各项规定，公道正派，谦虚谨慎，老老实实做人，干干净净做事，树立良好的人民公仆形象。

各位代表！做好今年各项工作，任务艰巨，责任重大。让我们在自治区党委、政府和市委的坚强领导下，团结和带领全市270万各族人民，解放思想，扎实工作，锐意改革，开拓创新，为实现呼伦贝尔"美丽发展、富民强市"的宏伟目标而努力奋斗！
